# From Sprint to Endurance: Performance Level and Pacing Profile of International Level Para‐Cyclists From C Division

**DOI:** 10.1002/ejsc.12277

**Published:** 2025-04-09

**Authors:** Bryan Le Toquin, Mélanie Baconnais, Imad Hamri, Nicolas Forstmann, Thierry Weissland, Jean‐François Toussaint, Julien Schipman

**Affiliations:** ^1^ Institut de Recherche Bio‐Médicale et d’Épidémiologie du Sport (IRMES) UPR 7329, Institut National du Sport, de l’Expertise et de la Performance (INSEP) Paris France; ^2^ Université de Paris Paris France; ^3^ Federation Française Handisport Paris France; ^4^ Centre d’Investigations en Médecine du Sport (CIMS) Hôtel‐Dieu, Assistance Publique ‐ Hôpitaux de Paris Paris France

**Keywords:** classification, cycling, pacing profile, para‐cycling, paralympic, performance

## Abstract

We investigated how para‐cycling sport classes in the C division influence performance levels and pacing profiles in track and road races compared to able‐bodied cyclists. For track races, we analyzed data from seven UCI World Para‐Cycling Track Championships and UCI World Track Championships (2014–2023) in the male's 1 km time trial and female's 500 m time trial events. Principal component analysis and hierarchical clustering were applied to 125 m splits to identify performance patterns among para‐cyclists. For road races, we examined data from individual time trials in 16 UCI World Para‐Cycling Cups and Championships (2014–2023) to compare mean speeds and pacing profiles across sport classes. Para‐cyclists in the C division performed significantly worse than able‐bodied cyclists in both male's 1 km and female's 500 m track time trials (*p* < 0.05). The analysis revealed a statistically significant variation in performance across sport classes for both track and road events (*p* < 0.05). However, when comparing adjacent classes, specifically M/WC1–C2 and M/WC3–C4, no significant differences were observed on the track (*p* > 0.05). Regarding pacing profiles, male MC2 and female WC5 athletes exhibited a pacing pattern characterized by a faster finish (*p* ≤ 0.01). In the individual time trial, MC3 had a lower mean speed in the second and third laps than in the first lap (*p* ≤ 0.01), whereas MC4 and MC5 showed no significant mean speed differences across the three laps (*p* ≥ 0.05). This study demonstrates that performance levels and pacing profiles are sport‐class specific and event dependent.

**Summary**

- Performance levels and pacing profiles vary significantly across sport classes and are event specific.
- Male MC2 para‐cyclists are more impacted by the standing start in the 1 km time trial.
- Male MC1, MC2, and MC3 para‐cyclists experience a decrease in speed during the individual time trial, whereas MC4 and MC5 maintain a consistent pacing profile.

## 1 Introduction

Ensuring fair competition is one of the most significant challenges faced by the International Paralympic Committee (IPC) in parasports. According to the classification code, this can be achieved “by minimizing the impact of an individual's impairment on the outcome so that sporting ability, skill level, and training alone determine success and the final result” (S. Tweedy et al. 2011). Thus, an evidence‐based classification system is essential. The classification process involves a detailed assessment of an athlete's impairment to determine the appropriate tests for classification. Such a system relies on empirical evidence to establish the relationship between impairment and sports performance (S. Tweedy et al. 2009). Para‐cycling comprises four distinct divisions: cycling (C), handcycle (H), tandem (B), and tricycle (T) (Union Cycliste Internationale, 2020). Athletes are assigned to one of these divisions based on the nature of their impairment. Within each division, athletes are further classified into sport classes according to the severity of their impairment (Table 1) (UCI 2023). This article focuses exclusively on the C division. In recent years, studies aimed at providing robust scientific data to support performance factors in Paralympic sports have increased, particularly in elite Paralympic performance (Schipman et al. 2021; Hogarth et al. 2021; Le Toquin et al. 2021). Specifically, para‐cycling has seen a rise in research contributing to the identification of sport‐specific demands and improvements in the classification system (J. B. Liljedahl et al. 2020; Borg et al. 2022; Muchaxo et al. 2020). Para‐cycling stands out among parasports as a discipline that highlights the complex interaction between an athlete's impairments and their abilities due to the multiple timed events and their unique mechanical and physiological demands. Studies have demonstrated hierarchical performance differences between sport classes, although some adjacent sport classes exhibit similar performance levels (J. B. Liljedahl et al. 2020; Borg et al. 2021). These findings are particularly relevant within the C division, where, during the Paralympic Games, the C1–C2–C3 and C4–C5 sport classes are combined for specific events (e.g., the 1 km track time trial and road mass start race), with a time factor applied to track events. Although this grouping addresses practical concerns, such as the limited number of participants in the more severely impaired sport classes, the selection of sport‐class combinations and the calculation of time factors remain underexplored.

**TABLE 1:** Description of the impairments that constitute up C division sport class. More details on UCI rules and regulation; Part 16 para‐cycling; 01.08.2023 https://assets.ctfassets.net/761l7gh5x5an/4It6Z1SyGTtc9VByrfHbB6/e041f78adf6b5284f449aea8650e007f/16‐PAR‐20230801‐F.pdf.

Sport‐class	Descriptions
C1	Include moderate quadriplegia, multiple amputations, severe cerebral palsy, and severe neuromuscular disorders. Impairments affecting balance, coordination, and strength. Significant bike modifications required
C2	Include mild paraplegia, unilateral or bilateral amputations, and moderate hemiplegia. Impairments with better coordination than C1. Bike adjustments possible for balance compensation
C3	Include amputation of one upper or lower limb, moderate cerebral palsy, and neuromuscular disorders causing partial weakness. Minor bike adaptations (pedals, handlebars, and saddle)
C4	Include above‐knee or above‐elbow amputation, mild hemiparesis, and moderate musculoskeletal disorders affecting balance or muscle strength. Minimal bike adjustments
C5	Include amputation of a hand or foot with minor functional limitations and mild musculoskeletal disorders. Minimal impairment with little impact on balance or strength. Minor or no adaptations required

Pacing, defined as the goal‐directed distribution and management of effort throughout an exercise bout (Edwards and Polman 2012), is a crucial aspect of both para‐cycling and cycling performance (Corbett 2009; De Larochelambert et al. 2020; Wright 2016). The pacing profile, which refers to the evolution of speed at each split, provides essential insights into the physiological processes occurring during such events (Corbett 2009). In Paralympic sports and para‐cycling, it can also offer valuable information on the impact of impairment on performance (Wright 2016; Runciman et al. 2016). Studies have shown that male and female C1 to C3 sport classes exhibit significant speed differences associated with the severity of impairment during the 1 km time trial (1 km TT) at the World Para‐Cycling Championships and the 2012 London Paralympics (Wright 2016; Leprêtre et al. 2012). The impact of impairment on pacing profiles has also been highlighted. Athletes in the C1–C2 sport classes tend to have lower starting speeds, primarily due to the nature of their impairments (e.g., hemiplegia, diplegia, and arm and leg amputations). Such impairments require athletes to take longer to reach maximum power output and, consequently, their peak speed (Wright 2016; Leprêtre et al. 2012). These studies represent the first steps in understanding the relationship between impairment, performance, and pacing profiles. However, pacing profiles have not yet been investigated in other sport classes within the C division or across different events for male and female athletes.

The 1 km time trial (TT) for male and the 500 m TT for female are track events contested by both able‐bodied and para‐cyclists. These events are classified as sprint races, that is, explosive time trial events over a short distance (Stadnyk et al. 2022). Able‐bodied and para‐cyclists typically complete the 1 km TT in about 1 minute, highlighting the high intensity and speed required for this event. The metabolic response primarily relies not only on phosphocreatine, phosphorylation, and glycolysis but also on an athlete's functional capacities, particularly their ability to generate high torque at the start (Stadnyk et al. 2022). Comparing able‐bodied and para‐cyclists in this shared event provides valuable insights into the impact of impairment. In the study by Wright et al., a similar “all‐out” pacing profile was identified in both male and female able‐bodied and para‐cyclists. However, able‐bodied cyclists completed a lower proportion of their overall time in the initial 125 m split, indicating a faster start compared to para‐cyclists. In the final stages of the race, able‐bodied cyclists spent a greater proportion of time in the subsequent race splits, demonstrating a more pronounced decline in speed (Wright 2016). On the road, the individual time trial (ITT) in para‐cycling, which typically lasts around 30 min, is considered an endurance event. This classification is based on the predominant reliance on the aerobic energy system over anaerobic contribution, emphasizing endurance performance factors such as maximal oxygen consumption, lactate threshold, and efficiency/economy (Coyle 1999). Analyzing this event and its execution can provide insights into the physiological impact of impairment. Thus, this study aims to examine how sport classes influence performance levels and pacing profiles in C‐division para‐cyclists across both sprint and endurance events in male and female athletes.

## 2 Methods

### 2.1 Data Collection

Publicly available data were extracted from the RSSTiming (https://www.rsstiming.com) and TissotTiming (https://www.tissottiming.com) websites. The data correspond to C1 to C5 sport classes based on the IPC para‐cycling classification system (Cycling Classification). The prefixes M and W denote male and female sport classes, respectively (e.g., MC1 or WC1), whereas the letter ‘C' without prefixes is used in the manuscript when referring to both male and female.

#### 2.1.1 1 km Time Trial/500 Time Trial Data

In this article, the 1 km time trial (1 km TT) will always refer to the male's race and the 500 m time trial (500 m TT) to the female's race. Finishing times and 125 m split times were collected from seven UCI World Para‐Cycling Track Championships and UCI World Track Championships for able‐bodied cyclists (ABC) between 2014 and 2023, including only events where split time data were available. The best individual time for each athlete, along with the corresponding split times, was retained.

#### 2.1.2 Individual Time Trial Data

Data were collected from individual time trials (ITTs) held at nine male's and seven female's World Para‐Cycling Cups and World Para‐Cycling Championships between 2014 and 2023, including only events where the lap course was identical and lap time data were available.

The performance analysis was divided into two parts:

- The first part corresponds to events where MC1, MC2, and MC3 as well as WC1 to WC5 covered the same distance over two laps.
- The second part corresponds to events where MC3, MC4, and MC5 covered the same distance over three laps.

### 2.2 Statistical Analysis

#### 2.2.1 1 km Time Trial/500 m Time Trial Races

To assess pacing profiles and performance level classes at each 125 m split among para‐cyclists, principal component analysis (PCA) was performed separately for the 1 km time trial and the 500 m time trial. This method reduces dataset dimensionality while preserving as much variance as possible, facilitating a clearer interpretation of the data by identifying key variables that explain most of the variance within a coordinate system defined by two axes (Bro et al. 2014). Performance levels were derived from race times at each split and converted into mean speed (in km·h^−1^). To test the hypothesis of a difference between sport classes on each PCA component, a nonparametric Kruskal–Wallis test was conducted, as the homoscedasticity assumption was rejected using Levene's test. Pairwise comparisons of sport classes on each PCA component were then conducted using the Mann–Whitney post hoc test with Bonferroni correction. Assuming that one of the PCA components represents the pacing profile, we tested whether the mean coordinate of each sport class on this PCA component differed significantly from zero. This was done using a Student's *t*‐test, following verification of data normality and homoscedasticity. A mean significantly different from zero would indicate a distinct pacing profile, such as a faster start or finish, compared to the dataset. A hierarchical clustering analysis was performed on the coordinates obtained through PCA to identify similarities in performance and pacing profiles across sport classes. This clustering method iteratively merges data into larger clusters based on Euclidean distance. The positions and colors of points reflect their affiliation with a specific cluster. To test the hypothesis of a difference between clusters on each PCA component, two nonparametric Kruskal–Wallis tests were conducted, as the normality assumption was rejected using the Shapiro–Wilk test. Pairwise comparisons of clusters on each PCA component were then conducted using the Mann–Whitney post hoc test with Bonferroni correction. Finally, to analyze the distribution of para‐cyclists within the clusters, a contingency table was created and results were reported as percentages (%).

#### 2.2.2 Individual Time Trial Races

Mean speeds (in km·h^−1^) throughout each lap were compared across sport classes to identify performance variations and within sport classes to highlight pacing profile differences. For the intersport class comparison, mean speeds (km·h^−1^) between sport classes were analyzed using a nonparametric Friedman test, following confirmation of the data's abnormal distribution. A Dunn post hoc test was then applied to identify mean speed differences between adjacent sport classes. The intraclass analysis varied based on the number of comparisons. For two‐lap races within C1, C2, and C3, differences in mean speeds were assessed using the nonparametric Wilcoxon signed‐rank test. For three‐lap races, a nonparametric Friedman test was performed, following confirmation of the data's abnormal distribution with a Shapiro–Wilk test. In cases of significant differences, a Dunn post hoc test was conducted to identify differences between laps. Results are presented as mean speed (km·h^−1^) ± standard deviation. For all statistical analyses, the significance level was set at *α* = 0.05. Data processing, graphical visualization, and statistical analyses were performed in R (version 4.0.3; The R Foundation for Statistical Computing, Vienna, Austria).

## 3 Results

### 3.1 1 km Time Trial/500 m Time Trial

The track race dataset includes unique performances from 340 male and 134 female athletes (Table 2). WC1 was combined with WC2 due to the low number of athletes (*n* = 6). The principal component analysis (PCA) of the male's 1 km TT identified two major components that together explained 98.52% of the overall variance. The first component (PC1) accounted for 93.95% of the variance and was strongly associated with the athletes' performance levels throughout the race (Table 3). The second component (PC2), explaining 5.57% of the variance, represented the pacing profile of each athlete (Table 3). Similarly, the PCA of the female's 500 m TT identified two major components, explaining 99.6% of the overall variance (PC1: 97.00% and PC2: 2.60%). As observed in the male's 1 km TT, PC1 in the female's event was strongly associated with the performance levels of para‐cyclists, whereas PC2 reflected their pacing profiles (Table 3).

**TABLE 2:** Number of male and female performances (*n*), mean speeds (km h^−1^ ± sd), and mean distance (km ± sd) in 1 km/500 m time trial and individual time trial by sport‐class and gender.

1 km time trial/500 m time trial			Individual time trial					
			2 laps			3 laps		
	*n*	Mean speed (km h^−1^)	*n*	Mean speed (km h^−1^)	Mean distance (km)	*n*	Mean speed (km h^−1^)	Mean distance (km)
Male								
MC1	27	46.62 ± 2.96	45	36.8 ± 1.91	18.52 ± 0.91			
MC2	53	48.02 ± 4.17	74	37.6 ± 2.56	18.52 ± 0.91			
MC3	47	51.92 ± 3.69	70	40.2 ± 2.04	18.52 ± 0.91	97	40.3 ± 3.56	28.8 ± 2.70
MC4	54	52.75 ± 3.04				91	41.5 ± 2.81	28.8 ± 2.70
MC5	43	55.03 ± 2.89				97	41.6 ± 4.02	28.8 ± 2.70
Able‐bodied	50	59.57 ± 1.76						
Female								
WC1–C2	28	46.81 ± 4.96	76	32.98 ± 3.80	18.35 ± 1.65			
WC3	15	50.02 ± 2.80	49	36.41 ± 2.60	18.35 ± 1.65			
WC4	32	50.87 ± 3.66	69	36.12 ± 3.41	18.25 ± 1.65			
WC5	29	53.99 ± 3.07	85	37.88 ± 3.07	18.25 ± 1.65			
Able‐bodied	30	61.98 ± 1.16						

**TABLE 3:** Results of principal component analysis (PCA). Coordinate of each variable in 1 km time trial and 500 m time trial in each PCA components as well as percentage of variance.

	Principal component 1 (PC1)	Principal component 2 (PC2)
Male 1 km time trial		
Variance (in %)	93.95	4.57
Mean speed 125 m	0.912	0.361
Mean speed 250 m	0.968	0.232
Mean speed 375 m	0.984	0.132
Mean speed 500 m	0.992	0.023
Mean speed 625 m	0.994	−0.063
Mean speed 750 m	0.986	−0.155
Mean speed 875 m	0.973	−0.217
Mean speed 1000 m	0.942	−0.298
Female 500 m time trial		
Variance (in %)	97.001	2.603
Mean speed 125 m	0.970	0.235
Mean speed 250 m	0.995	0.049
Mean speed 375 m	0.996	−0.083
Mean speed 500 m	0.978	−0.199

**FIGURE 1:**
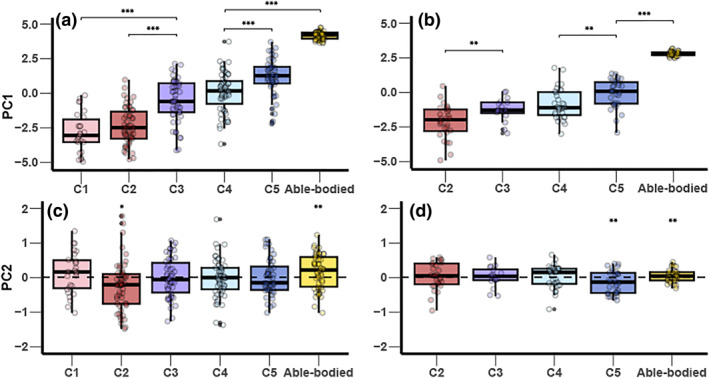
Boxplot of each para‐cyclist sport‐class and able‐bodied cyclist in 1 km time trial on PC1 (a) and PC2 (c) and 500 m time trial on PC1 (b) and PC2 (d). (*Indicate a significant difference between sport‐class (*p* ≤ 0.05); **indicate a significant difference between sport‐class (*p* ≤ 0.01); and ***indicate a significant difference between sport‐class (*p* ≤ 0.001)).

**FIGURE 2:**
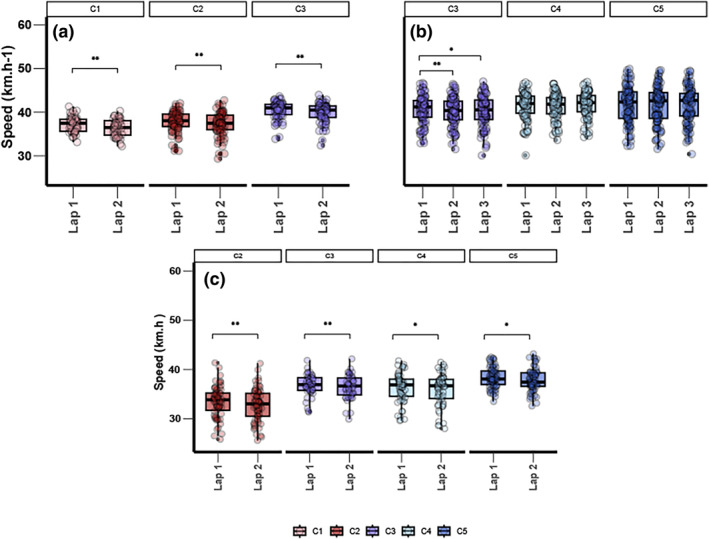
Mean speed (in km h^−1^) at each lap by sport‐class for male 2 laps races (a) and **3** laps races (b) and female 2 laps races (c). (**Indicate a significant mean speed difference between 2 laps (*p* ≤ 0.01) and ***indicate a significant mean speed difference between 2 laps (*p* ≤ 0.00).

A significant difference was observed across all sport classes on PC1 in the male's 1 km TT and female's 500 m TT (*p* ≤ 0.001) (Figure 1a,b). In both male's and female's events, post hoc testing revealed that C3 was faster than both C1 and C2 (*p* ≤ 0.01) but significantly slower than C5 and ABC (*p* ≤ 0.001). No statistically significant difference was found between MC1 and MC2 in the 1 km TT (*p* = 0.250), suggesting that the null hypothesis of equal medians could not be rejected. Similarly, no significant difference was detected between C3 and C4 in either event (*p* = 0.210 in 1 km TT and *p* = 0.580 in 500 m TT). C4 athletes were slower than those in C5 and ABC (*p* ≤ 0.01) in both male's and female's events. On PC2, MC2 and WC5 exhibited a pacing pattern indicative of a faster finish (*p* ≤ 0.05) (Figure 2a,b). Male's ABC were characterized by a faster start (*p* ≤ 0.05). No statistically significant differences were noted for other male's and female's sport classes on PC2 (*p* ≥ 0.05), indicating a relatively neutral pacing profile.

The clustering analysis based on PCA components revealed three distinct clusters among male and female para‐cyclists in each event. On PC1, significant differences were observed between all clusters in both events (*p* ≤ 0.001). On PC2, no significant differences were detected between clusters in either event (*p* ≥ 0.05), indicating that the null hypothesis of equal medians could not be rejected. The distribution of sport classes within each cluster is shown in Table 4. In the 1 km TT, Cluster 1 was primarily composed of MC1 (81.48%) and MC2 (75.93%) para‐cyclists. Cluster 2 mainly included MC3 (74.00%), MC4 (73.21%), and MC5 (74.47%). Finally, Cluster 3 consisted exclusively of ABC (100%). In the 500 m TT, Cluster 1 was predominantly made up of WC1–WC2 (89.25%), with a high proportion of WC3 (62.50%) and WC4 (56.25%). Cluster 2 primarily included WC5 (83.34%), along with 34.50% of WC2 and WC3. Cluster 3 consisted exclusively of female able‐bodied cyclists (100%).

**TABLE 4:** Contingency table representing the percentages of para‐cyclists and able‐bodied cyclists within each cluster (in %). Bold values highlight the cluster where para‐cyclists are in the largest proportion.

	Cluster 1	Cluster 2	Cluster 3
**Male**			
MC1	**81.48**	18.52	0.00
MC2	**75.93**	24.07	0.00
MC3	26.00	**74.00**	0.00
MC4	19.64	**73.21**	7.15
MC5	6.38	**74.47**	19.15
Able‐bodied	0.00	0.00	**100.00**
**Female**			
WC1–C2	**89.25**	10.72	0.00
WC3	**62.50**	37.50	0.00
WC4	**56.25**	37.50	6.25
WC5	16.66	**83.34**	0.00
Able‐bodied	0.00	0.00	**100.00**

### 3.2 Individual Time Trial

The individual time trial dataset includes 543 male and 279 female performances. For male's intersport class comparisons, a significant difference in mean speed was observed between MC1, MC2, and MC3 in each of the two laps (*p* ≤ 0.01). Post hoc tests revealed significant mean speed differences between MC1 and MC2, MC1 and MC3, and MC2 and MC3 in each lap (*p* ≤ 0.01). In male's three‐lap races, a significant difference in mean speed was observed between MC3 and MC4 in each lap (*p* ≤ 0.01). In the first lap, the post hoc test indicated differences between MC4 and MC5 (*p* ≤ 0.01). No significant difference was found between MC3 and MC4 (*p* ≥ 0.05). In the second and third laps, post hoc tests revealed significant mean speed differences between MC3 and MC4 and between MC4 and MC5 (*p* ≤ 0.05). For female's intersport class comparisons, a significant difference in mean speed was observed between WC2 and WC3 and between WC4 and WC5 (*p* ≤ 0.01) in each lap. No significant difference was detected between WC3 and WC4 (*p* = 0.18) in any lap.

For male's two‐lap races, intrasport class analysis showed that MC1, MC2, and MC3 had faster mean speeds in the first lap than in the second (*p* ≤ 0.01) (Figure 2a). In male's three‐lap races, MC3 had a lower mean speed in the second and third laps than in the first lap (*p* ≤ 0.01). MC4 and MC5 showed no significant differences in mean speed across the three laps (Figure 2b). For female's two‐lap races, intrasport class analysis showed that WC2, WC3, WC4, and WC5 had faster mean speeds in the first lap than in the second (*p* ≤ 0.01) (Figure 2c).

## 4 Discussion

This study is the first to investigate the association between pacing profiles and performance among male and female para‐cyclists across all sport classes in the C division. It aims to examine how sport classes within the C division influence performance levels and pacing profiles, from a track sprint race (1 km TT and 500 m TT) to an endurance race in road conditions (ITT), through a comparison with able‐bodied cyclists. Our findings indicate that: (1) Para‐cyclists in the C division perform at a significantly lower level than able‐bodied cyclists in both male's and female's 1 km and 500 m TT, whereas C1–C2 and C3–C4 exhibit similar performance levels. (2) Male MC2 and female WC5 display distinct pacing profiles in track 1 km and 500 m TT. (3) Performance levels in ITT follow a hierarchical structure between C1 and C5. (4) MC1, MC2, and MC3 exhibit a positive pacing profile, whereas MC4 and MC5 maintain a steady pacing profile in road ITT.

### 4.1 Track 1 km and 500 m Time Trial

The first result highlights that performance levels in the para‐cycling C division were significantly lower than those of able‐bodied cyclists, with a hierarchical structure observed from C1 to C5. This finding aligns with previous studies comparing para‐cyclists with able‐bodied cyclists (J. B. Liljedahl et al. 2020; Wright 2016). The disparity in performance levels across sport classes may be attributed to varying degrees of muscle strength limitations. The C division includes athletes with moderate to severe hemiplegia, diplegia, or ataxia as well as those with leg and arm amputations, incomplete spinal cord injuries, or muscular impairments (UCI 2023). Research on individuals with cerebral palsy (CP), para‐athletes with hemiplegia, and amputee cyclists suggests that muscle strength, lower limb power, torque production, and balance limitations are key factors affecting lower extremity performance (Brickley et al. 2010; P. Runciman et al. 2015; Dyer 2016; Reina et al. 2020). The analysis also revealed similar performance levels between adjacent sport classes, specifically between MC1–MC2 and C3–C4, in both male and female categories. These results differ slightly from Liljedahl's study, which found significant performance differences across all sport classes in the C division. This discrepancy may be due to a statistical effect related to the study population, as Liljedahl's analysis included only the top five athletes in each sport class. The absence of significant differences between adjacent categories does not challenge the validity of the athlete classification system. However, it may indicate the need for more comprehensive and targeted investigations into the effects of impairment when comparable performance levels are observed between adjacent categories in this specific event. The C3 and C4 sport classes include athletes with tibial amputations who rely on prostheses. It remains uncertain whether these athletes have an advantage over other para‐cyclists within the same class who do not use such equipment. Further research is needed to examine how technological innovations, particularly advancements in prosthetics, may influence performance. Such investigations are essential for informing sport‐specific regulations. Additionally, athletes can use bicycles equipped with aero bars, which reduce the rider's frontal area, thereby decreasing aerodynamic drag—a factor that accounts for up to 90% of resistance at para‐cycling speeds in track events (Grappe et al. 1997). The use of aero bars increases an athlete's speed for the same power output. However, due to their impairment, some para‐cyclists in the C1–C3 sport classes may experience balance issues when using aero bars. Wright et al. used video analysis of the 2012 London Paralympic Games and observed that not all athletes were able to use aero bars. Therefore, the use of aero bars could contribute to variations in performance (Wright 2016).

Our 1 km TT data indicate that male able‐bodied cyclists adopt a fast‐start profile compared to para‐cyclists in the C division. This profile is characteristic of an all‐out pacing strategy, corroborating previous findings by Wright et al., which demonstrated that able‐bodied cyclists exhibit a fast start followed by a significant decay in speed. This pattern is primarily attributed to the functional limitations present in all para‐cycling sport classes (Wright 2016). Our study reveals a distinct pacing pattern in MC2 and WC5. MC2 athletes are characterized by a slower start but a relatively faster finish. They require more time to reach their maximum speed but can maintain it until the end of the 1 km TT. These findings confirm previous observations showing that C2 para‐cyclists were slower than C3 at the start of the 1 km TT (Wright 2016). This result can likely be attributed to delays in reaching peak power and higher mechanical and metabolic loads, particularly when using only one limb (Wright 2016; Leprêtre et al. 2012; Bundle et al. 2006). Factors, such as asymmetrical torque production, balance, and coordination limitations, could also impact an athlete's ability to effectively launch from the starting block (Dyer 2016; Reina et al. 2020). Furthermore, the inability of multiple athletes in these sport classes to transition into a standing position can further reduce peak power output (Reiser et al. 2002). C3, C4, and C5 para‐cyclists exhibit a distinct pacing strategy compared to C1 and C2, characterized using a more neutral pacing approach. Their effort is not marked by an explosive start or a strong finish in contrast to other sport classes or able‐bodied cyclists. The lesser impact of impairment on torque production, allowing them to use larger gear ratios, as well as their better balance and ability to start in a standing position, could explain these differences. In the female's 500 m TT, only WC5 exhibited a specific pacing profile, characterized by a faster finish. Compared to other sport classes, their less severe functional limitations, particularly their greater torque capacity, enable them to use larger gear ratios, allowing them to continuously increase their speed throughout the effort. In contrast, other female sport classes reach their peak speed earlier in the 500 m TT. More studies are needed to gain deeper insight into the specific impact of impairments on sprint cycling performance as well as potential sex differences in pacing strategies.

### 4.2 Clustering Analysis

**FIGURE 3:**
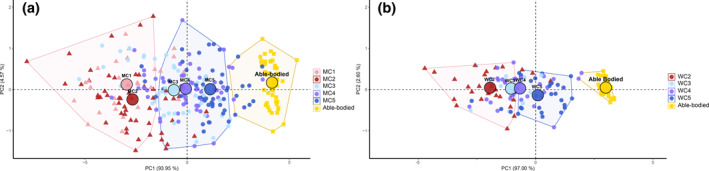
Clustering analysis in 1 km time trial (a) and 500 m time trial (b). The 3 clusters are formed using the colored polygons. Each dot represents all para‐cyclists and able‐bodied cyclists. Dots shape are unique for each sport‐class. Dots in black outlines are the barycenter of each sport‐class.

The clustering analysis predominantly grouped MC1–MC2 in Cluster 1, MC3–MC5 in Cluster 2, and male able‐bodied cyclists in Cluster 3 (Figure 3). Meanwhile, WC1/WC2, WC3, and WC4 were grouped in the same cluster (Figure 3). Clustering was primarily based on the performance level of para‐cyclists. These results must be interpreted in light of the sport class combinations implemented in the Paralympic Games. The C1–C3 sport classes are combined, as are C4 and C5, in the 1 km TT, 500 m TT, and road mass start races. This classification system has major implications for athlete selection at the Paralympic Games. On the track, a time factor is applied to performance times to ensure fairness, with variations across different classification groups. Based on our results—and the closer performance levels observed between C3, C4, and C5 in the 1 km TT and 500 m TT—we suggest that re‐evaluating sport‐class combinations may be warranted. However, further investigation is needed to determine whether the current sport‐class groupings and the time factor adjustments create a truly fair system for all para‐cyclists. Furthermore, the cluster analysis method based on PCA offers a valuable approach for studying the impact of classification on performance factors. This method enables the identification of athletes who outperform their assigned sport class, allowing for an assessment of whether certain impairment profiles may provide unintended advantages in specific events.

For instance, a classification challenge highlighted by a Delphi study (J. Liljedahl 2022) concerns the inclusion of one‐legged para‐cyclists in the same sport class as athletes with limited but functional use of both legs. In a track event, such as the 1 km TT/500 m TT, which involves a stationary start, it could be hypothesized that one‐legged para‐cyclists face greater power production limitations than hemiplegic athletes, making this an important factor influencing performance outcomes. The next step in this analysis could involve examining the distribution of athletes with unilateral leg impairments across clusters to confirm or refute this hypothesis among para‐cyclists. Additionally, this method could be applied across various Paralympic sports where multiple performance factors can be quantified.

### 4.3 Individual Time Trial Races

Our study also highlights differences based on the event considered. The analysis of ITT confirms existing literature showing significant performance differences between each adjacent sport class (Borg et al. 2022), which slightly contrasts with the 1 km TT and 500 m TT events. In ITT, differences in performance and pacing profiles were observed (Figure 2a). Male MC1, MC2, and MC3 as well as female WC1, WC2, WC3, WC4, and WC5 displayed a similar pacing profile characterized by a fast start followed by a decrease in speed. This pattern was observable for MC3 in both two‐lap and three‐lap races. However, different trends were noted in MC4 and MC5, which were characterized by a steady pacing profile throughout the race (Figure 2b). The decrease in speed throughout the race for male and female C1, C2, and C3 could be explained by the shorter race distance (Table 2), allowing athletes to adopt a more “all‐out” strategy. However, when the race distance for MC3 matches that of MC4 and MC5, the pacing profile still shows a decline in speed, whereas MC4 and MC5 athletes are able to maintain their speed throughout the race.

In an ITT, individual performances are significantly influenced by physiological capacities, as glycolytic and oxidative demands are essential for maintaining an intense effort over a short duration (Støren et al. 2013). Athletes in the C division may face physiological limitations that impact their ability to maintain a consistent level of effort during a longer event (Leprêtre et al. 2012). Literature is scarce on the physiological impact of impairments, but para‐cyclists from C1 to C3 sport classes can display both central and peripheral limitations compared to those in C4–C5. C1–C2 para‐cyclists with CP can face reduced venous return due to spasticity and decreased energy efficiency resulting from coordination deficits and pedaling asymmetry (Brickley et al. 2010; Prins et al. 2016; Kloyiam et al. 2011). Additionally, limitations in the engaged muscle mass in CP and amputee athletes can lead to an increase in blood lactate concentration and reduced metabolic efficiency (Goosey‐Tolfrey TP Vicky 2018). Also, depending on the location of the limb deficiency and the use of prosthetics, athletes can experience thermoregulation challenges, as prosthetics can act as insulators and lead to the accumulation of sweat (Griggs et al. 2020).

The congenital or acquired origin of an impairment might also be a factor to consider. The developmental pathways and physiological development of para‐cyclists could be impacted by the origin of their impairment and thus play an important role in the classification process as observed in other Paralympic sports (Le Toquin et al. 2021; Lopes‐Silva, Franchini, and Kons 2023). There is still a lack of research on the specific physiological responses to para‐cycling in relation to impairment, particularly in elite athletes. The differences in performance patterns between sprint races and road races highlight the need for scientific evidence on the characterization of competitive demands across all para‐cycling events and for further knowledge on the impact of impairment (functional and physiological) on these competitive demands.

Our results must be interpreted considering limitations. The lack of data for both track and road events (especially in female events) and the influence of race‐course characteristics (distance and elevation) on the outcomes cannot be overlooked.

### 4.4 Perspectives

Future research should focus on investigating the physiological and biomechanical limitations associated with different types of impairments. Such analyses could provide a deeper understanding of how these factors shape pacing strategies and overall performance across different para‐cycling events. Moreover, extending this investigation to other divisions (B, H, and T) in road events, incorporating a larger data sample, and considering event‐specific factors, such as elevation and course length, would enhance the evidence base for the current classification system.

## 5 Conclusion

This study provides valuable insights into the performance and pacing profiles in para‐cycling across the C division in the sprint‐to‐endurance continuum. The differences in performance levels and pacing profiles between sport classes, depending on the event, underline the significant impact of physiological and functional limitations on the specific demands of para‐cycling. The close performance levels between adjacent sport classes in the track 1 km/500 m TT and the differences in pacing profiles between C1,C2, andC3 sport classes in road ITT emphasize the need for continued research on the specificities of para‐cycling.

## Ethics Statement

This study was designed and monitored by the Institut de Recherche bio‐Médicale et d’Epidémiologie du Sport (IRMES) scientific committee. Data collection was compliant with the General Data Protection Regulations applied in the European Union. A declaration of the study was made and approved by the Commission Nationale de l’Informatique et des Libertés (CNIL) with the following registration number: 2216887v0.

## Conflicts of Interest

The authors declare no conflicts of interest.

## Data Availability

Publicly available data were analyzed in this study. Data can be found here: https://www.tissottiming.com; https://www.rsstiming.com.

## References

[ejsc12277-bib-0001] Borg, D. N. , J. O. Osborne , S. M. Tweedy , J. B. Liljedahl , and C. F. J. Nooijen . 2021. “Bicycling and Tricycling Road Race Performance in International Para‐Cycling Events Between 2011 and 2019.” American Journal of Physical Medicine and Rehabilitation 101, no. 4: 384–388: Publish Ahead of Print. 10.1097/PHM.0000000000001819. 34121066

[ejsc12277-bib-0002] Borg, D. N. , J. O. Osborne , S. M. Tweedy , J. B. Liljedahl , and C. F. J. Nooijen . 2022. “Bicycling and Tricycling Road Race Performance in International Para‐Cycling Events Between 2011 and 2019.” American Journal of Physical Medicine and Rehabilitation 101, no. 4: 384–388. 10.1097/phm.0000000000001819. 34121066

[ejsc12277-bib-0003] Brickley, G. , and H. Gregson . 2010. “A Case Study on Torque Production of a Cerebral Palsy Paralympic Cyclist.” Journal of Science and Medicine in Sport 12: e104. 10.1016/j.jsams.2009.10.214.

[ejsc12277-bib-0004] Bro, R. , and A. K. Smilde . 2014. “Principal Component Analysis.” Analytical Methods 6, no. 9: 2812–2831. 10.1039/c3ay41907j.

[ejsc12277-bib-0005] Bundle, M. W. , C. L. Ernst , M. J. Bellizzi , S. Wright , and P. G. Weyand . 2006. “A Metabolic Basis for Impaired Muscle Force Production and Neuromuscular Compensation During Sprint Cycling.” American Journal of Physiology ‐ Regulatory, Integrative and Comparative Physiology 291, no. 5: R1457–R1464. 10.1152/ajpregu.00108.2006. 16840656

[ejsc12277-bib-0006] Corbett, Jo . 2009. “An Analysis of the Pacing Strategies Adopted by Elite Athletes During Track Cycling.” International Journal of Sports Physiology and Performance 4, no. 2: 195–205. 10.1123/ijspp.4.2.195. 19567923

[ejsc12277-bib-0007] Coyle, E. F. 1999. “Physiological Determinants of Endurance Exercise Performance.” Journal of Science and Medicine in Sport 2, no. 3: 181–189. 10.1016/s1440-2440(99)80172-8. 10668757

[ejsc12277-bib-0008] Cycling Classification & Categories.” International Paralympic Committee. 2023. [date unknown]; [cited 2023 Oct 16 ] https://www.paralympic.org/cycling/classification.

[ejsc12277-bib-0009] De Larochelambert, Q. , S. Del Vecchio , A. Leroy , S. Duncombe , J.‐F. Toussaint , and A. Sedeaud . 2020. “Body and Boat: Significance of Morphology on Elite Rowing Performance.” Frontiers in Sports and Active Living 2: 2. 10.3389/fspor.2020.597676. 33345179 PMC7739618

[ejsc12277-bib-0010] Dyer, B. 2016. “Cycling With an Amputation: A Systematic Review.” Prosthetics and Orthotics International 40, no. 5: 538–544. 10.1177/0309364615610659. 26527756

[ejsc12277-bib-0011] Edwards A. , Polman R. 2012. “Pacing in Sport and Exercise: A Psychophysiological Perspective.”

[ejsc12277-bib-0012] Goosey‐Tolfrey TP Vicky . 2018. “Applying Strength and Conditioning Practices to Athletes With a Disability.” Routledge Handbook of Strength and Conditioning. Routledge.

[ejsc12277-bib-0013] Grappe F. , Candau R. , Belli A. , Rouillon J. D. 1997. “Aerodynamic Drag in Field Cycling With Special Reference to the Obree’s Position.” Ergonomics 40, no. 12: 1299–1311. 10.1080/001401397187388.

[ejsc12277-bib-0014] Griggs, K. E. , B. T. Stephenson , M. J. Price , and V. L. Goosey‐Tolfrey . 2020. “Heat‐related Issues and Practical Applications for Paralympic Athletes at Tokyo 2020.” Temperature 7, no. 1: 37–57. 10.1080/23328940.2019.1617030. PMC7053936 32166104

[ejsc12277-bib-0015] Hogarth, L. , V. Nicholson , C. Payton , and B. Burkett . 2021. “Modelling the Age‐related Trajectory of Performance in Para Swimmers With Physical, Vision and Intellectual Impairment.” Scandinavian Journal of Medicine and Science in Sports 31, no. 4: 31–935. 10.1111/sms.13910. 33345411

[ejsc12277-bib-0016] Kloyiam, S. , S. Breen , P. Jakeman , J. Conway , and Y. Hutzler . 2011. “Soccer‐Specific Endurance and Running Economy in Soccer Players With Cerebral Palsy.” Adapted Physical Activity Quarterly 28, no. 4: 354–367. 10.1123/apaq.28.4.354. 21914907

[ejsc12277-bib-0017] Leprêtre, P.‐M. , T. Weissland , J. Slawinski , and P. Lopes . 2012. “Para‐Cycling Performance Was rather Limited by Physiological Than Functional Factors.” Frontiers in Physiology 3: [cited 2020 Sep 17]. 10.3389/fphys.2012.00327. PMC3429095 22934075

[ejsc12277-bib-0018] Le Toquin, B. , J. Schipman , Q. Larochelambert , G. Saulière , S. Duncombe , and J.‐F. Toussaint . 2021. “Is the visual impairment origin a performance factor? Analysis of international‐level para swimmers and para athletes.” Journal of Sports Sciences 40: 1–9. 34847816 10.1080/02640414.2021.1999618

[ejsc12277-bib-0019] Liljedahl, J. B. 2022. “Para–Cycling Classification in the Bicycle and Tricycle Divisions: A Delphi Study.” In Towards Evidence‐Based Classification in Para‐Cycling. 10.1097/PHM.0000000000002972 41871137

[ejsc12277-bib-0020] Liljedahl, J. B. , A. Bjerkefors , A. Arndt , and C. F. J. Nooijen . 2020. “Para‐Cycling Race Performance in Different Sport Classes.” Disability and Rehabilitation 0, no. 0: 1–5. 10.1080/09638288.2020.1734106. 32174176

[ejsc12277-bib-0021] Lopes‐Silva, J. P. , E. Franchini , and R. Kons . 2023. “Para Powerlifting Performance: A Retrospective Analysis Considering Origin of Impairment, Sport‐Classification and Sex.” American Journal of Physical Medicine and Rehabilitation 103, no. 4: 356–362. 10.1097/PHM.0000000000002307. 37405959

[ejsc12277-bib-0022] Muchaxo, R. , S. Groot , L. Woude , T. Janssen , and C. Nooijen . 2020. “Do Handcycling Time‐Trial Velocities Achieved by Para‐Cycling Athletes Vary Across Handcycling Classes?.” Adapted Physical Activity Quarterly: Adapted Physical Activity Quarterly 37: 1–20. 33022652 10.1123/apaq.2019-0143

[ejsc12277-bib-0023] Prins, L. , P. C. M. Wolters , E. Casalino , et al. 2016. “An Elite Runner With Cerebral Palsy: Cost of Running Determines Athletic Performance.” South African Journal of Sports Medicine 28, no. 1: 27–29. 10.17159/2078-516x/2016/v28i1a1415.

[ejsc12277-bib-0024] Reina, R. , D. Barbado , C. Soto‐Valero , J. M. Sarabia , and A. Roldán . 2020. “Evaluation of the Bilateral Function in Para‐Athletes With Spastic Hemiplegia: A Model‐Based Clustering Approach.” Journal of Science and Medicine in Sport 23, no. 8: 710–714. 10.1016/j.jsams.2020.01.003. 31956044

[ejsc12277-bib-0025] Reiser, R. , J. Maines , J. Eisenmann , and J. Wilkinson . 2002. “Standing and Seated Wingate Protocols in Human Cycling. A Comparison of Standard Parameters.” European Journal of Applied Physiology 88, no. 1–2: 152–157. 10.1007/s00421-002-0694-1. 12436284

[ejsc12277-bib-0026] Runciman, P. , W. Derman , S. Ferreira , Y. Albertus‐Kajee , and R. Tucker . 2015. “A Descriptive Comparison of Sprint Cycling Performance and Neuromuscular Characteristics in Able‐Bodied Athletes and Paralympic Athletes With Cerebral Palsy.” American Journal of Physical Medicine and Rehabilitation 94, no. 1: 28–37. 10.1097/phm.0000000000000136. 24919082

[ejsc12277-bib-0027] Runciman, P. , R. Tucker , S. Ferreira , Y. Albertus‐Kajee , and W. Derman . 2016. “Paralympic Athletes With Cerebral Palsy Display Altered Pacing Strategies in Distance‐Deceived Shuttle Running Trials.” Scandinavian Journal of Medicine & Science in Sports 26, no. 10: 1239–1248. 10.1111/sms.12575. 26493357

[ejsc12277-bib-0028] Schipman, J. , G. Saulière , B. Le Toquin , et al. 2021. “Involvement in Multiple Race Events Among International Para and Non‐Disabled Swimmers.” Frontiers in Sports and Active Living 2: [cited 2021 Mar 12]. 10.3389/fspor.2020.608777. PMC7876089 33585812

[ejsc12277-bib-0029] Stadnyk, A. M. J. , F. M. Impellizzeri , J. Stanley , P. Menaspà , and K. M. Slattery . 2022. “Testing, Training, and Optimising Performance of Track Cyclists: A Systematic Mapping Review.” Sports Medicine 52, no. 2: 391–401. 10.1007/s40279-021-01565-z. 34591266 PMC8803767

[ejsc12277-bib-0030] Støren, Ø. , K. Ulevåg , M. H. Larsen , E. M. Støa , and J. Helgerud . 2013. “Physiological Determinants of the Cycling Time Trial.” Journal of Strength & Conditioning Research 27, no. 9: 2366–2373. 10.1519/jsc.0b013e31827f5427. 23238091

[ejsc12277-bib-0031] Tweedy, S. M. , and Y. C. Vanlandewijck . 2009. “International Paralympic Committee Position Stand – Background and Scientific Rationale for Classification in Paralympic Sport.” British Journal of Sports Medicine 45, no. 4: 259–269. 10.1136/bjsm.2009.065060. 19850575

[ejsc12277-bib-0032] Tweedy, S. M. , and Y. C. Vanlandewijck . 2011. “International Paralympic Committee Position Stand‐Background and Scientific Principles of Classification in Paralympic Sport.” British Journal of Sports Medicine 45, no. 4: 259–269. 10.1136/bjsm.2009.065060. 19850575

[ejsc12277-bib-0033] UCI . 2023. “UCI – Cycling Regulations.”: [cited 2023 Nov 11 ] Available from: https://assets.ctfassets.net/761l7gh5x5an/2hSKKwLFWuz8ApFjJHZVWf/d08c2a8ef5c4f7e07b0bab262f6b13c7/16‐PAR‐20230801‐E.pdf.

[ejsc12277-bib-0034] Wright, R. L. 2016. “Positive Pacing Strategies Are Utilized by Elite Male and Female Para‐Cyclists in Short Time Trials in the Velodrome.” Frontiers in Physiology 6: [cited 2023 Oct 12]. 10.3389/fphys.2015.00425. PMC4716664 26834643

